# Editorial: Psychiatric and Pharmacotoxicological Insights on Appearance and Performance Enhancing Drugs

**DOI:** 10.3389/fpsyt.2022.918482

**Published:** 2022-05-26

**Authors:** Simona Zaami, Annagiulia Di Trana, Óscar García-Algar, Enrico Marinelli, Francesco Paolo Busardò

**Affiliations:** ^1^Unit of Forensic Toxicology, Section of Legal Medicine, Department of Anatomical, Histological, Forensic, and Orthopedic Sciences, Sapienza University of Rome, Rome, Italy; ^2^Section of Legal Medicine, Department of Excellence of Biomedical Sciences and Public Health, Polytechnic University of Marche, Ancona, Italy; ^3^Neonatology Unit, Hospital Clinic-Maternitat, ICGON, BCNatal, Barcelona, Spain; ^4^Department of Medico-Surgical Sciences and Biotechnologies, “Sapienza” University of Rome, Rome, Italy

**Keywords:** appearance and performance enhancing drugs, substance abuse, psychiatric effects, anabolic androgenic steroids (AAS), Chemsex, GHB pharmacotoxicology

Since the very beginning of the twenty-first century, the popularity of the so-called Appearance and Performance Enhancing Drugs (APEDs) has been growing steadily, affecting virtually all age groups (1).

This heterogeneous class of substances includes both legal and illegal substances, all capable of enhancing physical or mental performances. The most popular APEDs are Anabolic Androgenic Steroids (AAS), synthetic derivatives of the natural sex hormones consumed not only to enhance athletic performances, but also to increase the muscle mass. Chemsex drugs may be considered APEDs as well, since they are intentionally or unintentionally administered to enhance sexual performances (e.g., erectile dysfunction agents and alkyl nitrites) or to increase sex-drive (e.g., cocaine, methamphetamine, synthetic cathinones, and synthetic cannabinoids and gamma hydroxybutyric acid, GHB) (2). Similarly, cognitive enhancers are diverted prescription drugs used in medical practice to treat specific cognitive deficits in mental diseases, such as attention deficit hyperactivity disorder, Alzheimer's disease or aging (3). The current society standards of perfection and mental performance seem to be the main reason behind the misuse of such substances.

It is worth bearing in mind that the illegal market of APED is extremely volatile and adaptable, not only to meet all the ever-changing needs of abusers, but also to create new market avenues designed to satisfy the rising demand. Not unlike the New Psychoactive Substances phenomenon, toxicological and forensic data should support the efforts aimed at constantly monitoring illicit markets, in order to promptly identify newly arising consumption dynamics and trends (4).

This Research Topic aims to provide updated studies, reports, reviews, and mini reviews on psychiatric and pharmacotoxicological effects of AAD, Chemsex drugs and cognitive enhancers and related health threats. Furthermore, chemo-analytical aspects of all APED classes have also been outlined. Indeed, the articles here included, shed light on this extraordinary challenge to scientists and health professionals operating in the field of psychiatric care for patients with substance abuse issues.

The psychological distress in AAD users was the main topic of the research study by Chegeni et al., who applied the multigroup latent class analysis to examine the possible correlation between aggression and psychological distress and AAD use. In particular, they aimed to shed a light on the possible difference between male and female AAD abusers, in a sample of 206 subjects, mostly females. According to the submitted questionnaire results, the authors individuated 5 sub-groups: low aggression mild distress users (39.06%), moderate direct aggression-mild indirect aggression moderate distress users (22.95%), moderate aggression distress users (18.64%), high aggression moderate distress users (7.63%). Interestingly, females outnumbered males in such subgroups.

Research data on APED effects on the personality traits were reviewed by Zaami et al. Interestingly, the linkage between several psychopathological disorders of unclear prevalence and APED consumption was commonly reported in the literature. The increase of APED abuse was also highlighted by the authors, raising the attention on this emerging public health concern. To this concern, the authors highly recommend phasing in prevention and intervention projects, prioritizing psychological support for people with substance use issues, depression and anxiety, and body image disorders.

Rosenberger et al. focused on the Chemsex practice incidence in German-speaking countries. To this concern, an online survey was carried out to investigate the psychotropic substances consumption, sexual and health related behaviors in relation to the socio-demographic information of more than 120 participants who met the criteria for Chemsex users. Remarkably, a significant degree of awareness about the problematic use of sexual and use behavior was reported, even though a minority showed different motivations. All the participants stated they had used protection to prevent the disease transmission, but the general need to be more informed on Chemsex-related issues was also clearly manifested.

GHB use in Chemsex settings as a performance-enhancing drug and its toxicological characterization was reviewed by Giorgetti et al. The authors performed a literature search to elucidate the underlying scientific reasons for GHB consumption as an APED and to expound upon abuse patterns and health risks arising from its consumption in Chemsex. The panel of desired performance-related effects was wider in real cases and epidemiological studies, suggesting that much has to be still discovered on GHB pharmacotoxicology.

In conclusion, this Research Topic was meant to produce updated studies and reviews centered around the psychiatric and psychological implications of APED consumption, hopefully raising awareness about this spreading phenomenon. In order to do that, it is of utmost importance to take stock and raise awareness as to the new daunting challenges looming on the horizons, particularly those arising from the changing avenues of trafficking through underground, impervious dark web illicit markets, where the criminal element is almost invisible, “floating in the ether,” with an almost immaterial guise. Future research and law enforcement therefore needs to be more adaptable and multidisciplinary than ever, in light of the fact that the COVID-19 pandemic has brought about different abuse dynamics that may largely elude the measures on which we have long relied to intercept and tackle the major public health threats posed by APEDs, novel psychoactive substances and illegal drugs overall.

## Author Contributions

All authors listed have made a substantial, direct, and intellectual contribution to the work and approved it for publication.

## Conflict of Interest

The authors declare that the research was conducted in the absence of any commercial or financial relationships that could be construed as a potential conflict of interest.

## Publisher's Note

All claims expressed in this article are solely those of the authors and do not necessarily represent those of their affiliated organizations, or those of the publisher, the editors and the reviewers. Any product that may be evaluated in this article, or claim that may be made by its manufacturer, is not guaranteed or endorsed by the publisher.
